# Sequence heterogeneity of the PenA carbapenemase in clinical isolates of *Burkholderia multivorans*

**DOI:** 10.1016/j.diagmicrobio.2018.06.005

**Published:** 2018-06-18

**Authors:** Scott A. Becka, Elise T. Zeiser, Steven H. Marshall, Julian A. Gatta, Kevin Nguyen, Indresh Singh, Chris Greco, Granger G. Sutton, Derrick E. Fouts, John J. LiPuma, Krisztina M. Papp-Wallace

**Affiliations:** a Research Service, Louis Stokes Cleveland Department of Veterans Affairs Medical Center, Cleveland, OH 44106, USA; b J. Craig Venter Institute (JCVI), Rockville, MD 20850, USA; c Department of Pediatrics and Communicable Disease, University of Michigan Medical School, Ann Arbor, MI 48103, USA; d Department of Medicine, Case Western Reserve University School of Medicine, Cleveland, OH 44106, USA; e Department of Biochemistry, Case Western Reserve University School of Medicine, Cleveland, OH 44106, USA

**Keywords:** *Burkholderia*, β-Lactamase, Sequencing

## Abstract

Multidrug-resistant gram-negative pathogens are a significant health threat. *Burkholderia* spp. encompass a complex subset of gram-negative bacteria with a wide range of biological functions that include human, animal, and plant pathogens. The treatment of infections caused by *Burkholderia* spp. is problematic due to their inherent resistance to multiple antibiotics. The major β-lactam resistance determinant expressed in *Burkholderia* spp. is a class A β-lactamase of the PenA family. In this study, significant amino acid sequence heterogeneity was discovered in PenA (37 novel variants) within a panel of 48 different strains of *Burkholderia multivorans* isolated from individuals with cystic fibrosis. Phylogenetic analysis distributed the 37 variants into 5 groups based on their primary amino acid sequences. Amino acid substitutions were present throughout the entire β-lactamase and did not congregate to specific regions of the protein. The PenA variants possessed 5 to 17 single amino acid changes. The N189S and S286I substitutions were most prevalent and found in all variants. Due to the sequence heterogeneity in PenA, a highly conserved peptide (18 amino acids) within PenA was chosen as the antigen for polyclonal antibody production in order to measure expression of PenA within the 48 clinical isolates of *B. multivorans*. Characterization of the anti-PenA peptide antibody, using immunoblotting approaches, exposed several unique features of this antibody (i.e., detected <500 pg of purified PenA, all 37 PenA variants in *B. multivorans*, and Pen-like β-lactamases from other species within the *Burkholderia cepacia* complex). The significant sequence heterogeneity found in PenA may have occurred due to selective pressure (e.g., exposure to antimicrobial therapy) within the host. The contribution of these changes warrants further investigation.

## 1. Introduction

The genus *Burkholderia* encompasses ~114 to 117 species that include human, animal, and/or plant pathogens as well as species that possess environmental benefits (e.g., endophytic *B. phytofirmans* can prevent onion rot) (Eberl and Vandamme, 2016). Based on advances in whole genomic sequencing and phylogenetic analysis, researchers proposed reorganizing the plant-beneficial-environmental species into new genera, *Paraburkholderia* and *Caballeronia*, while the human pathogens would remain within the *Burkholderia* genus (Dobritsa et al., 2017; Dobritsa and Samadpour, 2016; Eberl and Vandamme, 2016; Sawana et al., 2014). Discrepancies remain, however, as some species possess dual beneficial and pathogenic potential (*Paraburkholderia ginsengisoli* and *Paraburkholderia tropica*) (Deris et al., 2010; Marks et al., 2016). Thus, the taxonomic organization of these species remains controversial.

Focusing on the *Burkholderia* pathogens, 2 major groups exist, the *Burkholderia cepacia* complex (Bcc) and the *Burkholderia pseudomallei* complex (Bpc). Bcc species can cause infections (e.g., pneumonia) in immunocompromised persons or in individuals with cystic fibrosis (Abbott and Peleg, 2015; Chiappini et al., 2014; Gautam et al., 2011; Hanulik et al., 2013; Marson et al., 2015). Among the Bcc, *Burkholderia multivorans* and *Burkholderia cenocepacia* are the most prevalent species recovered from persons with cystic fibrosis in the United States. Within the Bpc, *B. pseudomallei* is capable of causing a necrotizing pneumonia known as melioidosis and is considered a potential bioweapon (Perumal Samy et al., 2017). Bcc and Bpc species are typically antibiotic resistant, rendering effective antimicrobial therapy of infections a challenge (Abbott and Peleg, 2015; Chiappini et al., 2014; Lipuma, 2010; Rhodes and Schweizer, 2016).

A major antibiotic resistance determinant present in all species of *Burkholderia* is an inducible class A β-lactamase of the Pen family (e.g., PenA). In 2009, Poirel et al. (2009) characterized several Pen β-lactamases present in Bcc and published the initial nomenclature for the Pen-like β-lactamase family. Since that time, the number of *Burkholderia* species has increased as has the number of Pen-type enzymes (Table 1). Expression of *bla*_pen_ genes is regulated by a LysR-type transcriptional regulator, PenR through a system analogous to AmpC/AmpR regulatory pathways present in members of the *Enterobacteriaceae* and in *Pseudomonas aeruginosa* (Dhar et al., 2018; Trepanier et al., 1997). In addition, each Pen-like β-lactamase possesses a different substrate profile (Papp-Wallace et al., 2013a; Poirel et al., 2009). PenA of *B. multivorans* possesses a very broad substrate profile that includes carbapenems and is capable of hydrolyzing β-lactamase inhibitors (i.e., clavulanic acid, sulbactam, and tazobactam). Here, we describe the identification of 37 novel PenA variants from *B. multivorans*. Moreover, using sequence analysis, we generated a polyclonal antibody using on an antigen that comprised an 18-amino-acid peptide from PenA that is able to detect all of the different PenA variants via immunoblotting.

## 2. Materials and methods

### 2.1. Strains

The *Burkholderia* spp. clinical isolates were from the *B. cepacia* Research Laboratory and Repository strain collection as previously described (Papp-Wallace et al., 2017b). The construction of *Escherichia coli* DH10B pBC SK(+)*bla*_penA_ and *E. coli* DH10B pBC SK(+)*bla*_penI_ was as described (Papp-Wallace et al., 2013b).

### 2.2. Whole-genome sequencing of B. multivorans

Genomic DNA was purified from the clinical *B. multivorans* isolates using the MasterPure^™^ gram-positive DNA purification kit (Epicentre Inc, Madison, WI) as recommended by the manufacturer. The genomes of 48 *B. multivorans* isolates were sequenced at JCVI by Illumina NextSeq (2 × 150 bp). Paired-end libraries were constructed using Illumina NexteraXT kits. Sequence reads were generated with a target average read depth of ~100-fold coverage. Sequence reads for each isolate were assembled individually using *SPAdes* (Bankevich et al., 2012) and annotated using National Center for Biotechnology Information’s (NCBI’s) Prokaryotic Genome Annotation Pipeline (Tatusova et al., 2016). Raw DNA sequence reads were submitted to the NCBI Sequence Read Archive, and annotated genomes were deposited in the GenBank whole-genome sequencing repository, which can be obtained within BioProject PRJNA434393. Clustal Ω from the European Bioinformatics Institute (EMBL-EBI) was used to create a multiple sequence alignment using the primary amino acid sequences of the 37 PenA variants and a phylogenetic tree (Li et al., 2015; McWilliam et al., 2013; Sievers et al., 2011).

### 2.3. PCR and DNA sequencing of bla_pen_ from Burkholderia spp

Overnight cultures of *B. cenocepacia* AU0583, *Burkholderia pyrrocinia* AU1114, *Burkholderia vietnamiensis* AU3578, *Burkholderia ambifaria* AU5203, *Burkholderia stabilis* AU9035, *Burkholderia dolosa* AU9336, *Burkholderia gladioli* AU1009, and *Burkholderia ubonensis* AU7314 carrying different *bla*_Pen_ genes were boiled for 10 minutes at 99 °C. Polymerase chain reaction (PCR) was conducted using 1 μL of boiled cells (containing the extracted DNA) with the PCR Master Mix (Promega) and primers generated to detect *bla*_penB_, *bla*_penC_, *bla*_penD_, *bla*_penE_, *bla*_penF_, *bla*_penG_, *bla*_penH_, and *bla*_penN_ (Supplementary Table 1). The PCR products were cleaned using QIAquick PCR Purification Kit (Qiagen) and sent to Molecular Cloning Laboratories for DNA sequencing. The resulting DNA sequence files were analyzed using DNAstar software, and the final nucleotide sequences were submitted to NCBI and assigned the following Genbank accession numbers: MG839177–MG839184. Peptide sequences from the different Pen-like β-lactamases were aligned using Clustal Ω as described above.

### 2.4. Immunoblotting

All strains were grown in lysogeny broth to log phase at an OD_600nm_ between 0.6 and 0.7. In addition, the *Burkholderia* spp. were treated with 1 mg/L imipenem for 2 hours to induce expression of *bla*_pen_. Subsequently, the cells were pelleted and lysed using stringent periplasmic fractionation to prepare crude extracts, as previously described (Papp-Wallace et al., 2012). These crude extracts and purified full-length PenA protein were subjected to sodium dodecyl sulfate–polyacrylamide gel electrophoresis and transferred to polyvinylidene difluoride membranes. The membranes were blocked in 5% nonfat dry milk in 20 mM Tris–Cl with 150 mM NaCl at pH 7.4 (TBS) for 1 hour and probed in 5% nonfat dry milk in TBS with 1 μg/mL of polyclonal anti–PenA-peptide antibody (which was raised in rabbits by New England Peptide (NEP) using a selected PenA 18 amino acid peptide as the antigen). Membranes were washed 5 times for 10 minutes with TBS with 0.05% Tween 20 (TBST), and for protein detection, blots were incubated for 1 hour in 1:5000 dilutions of antirabbit and antimouse secondary horseradish peroxidase–conjugated antibodies in 5% nonfat dry milk in TBS. Blots were washed 5 times for 10 minutes with TBST and developed using the ECL-Plus^™^ developing kit (GE Healthcare Life Sciences) or the SuperSignal West Femto Chemiluminescent Substrate (Thermoscientific) according to the manufacturers’ instructions. The Fotodyne Luminary/FX was used to capture images.

## 3. Results and discussion

### 3.1. The PenA carbapenemase of B. multivorans exhibits considerable sequence heterogeneity

**Fig. 1:**
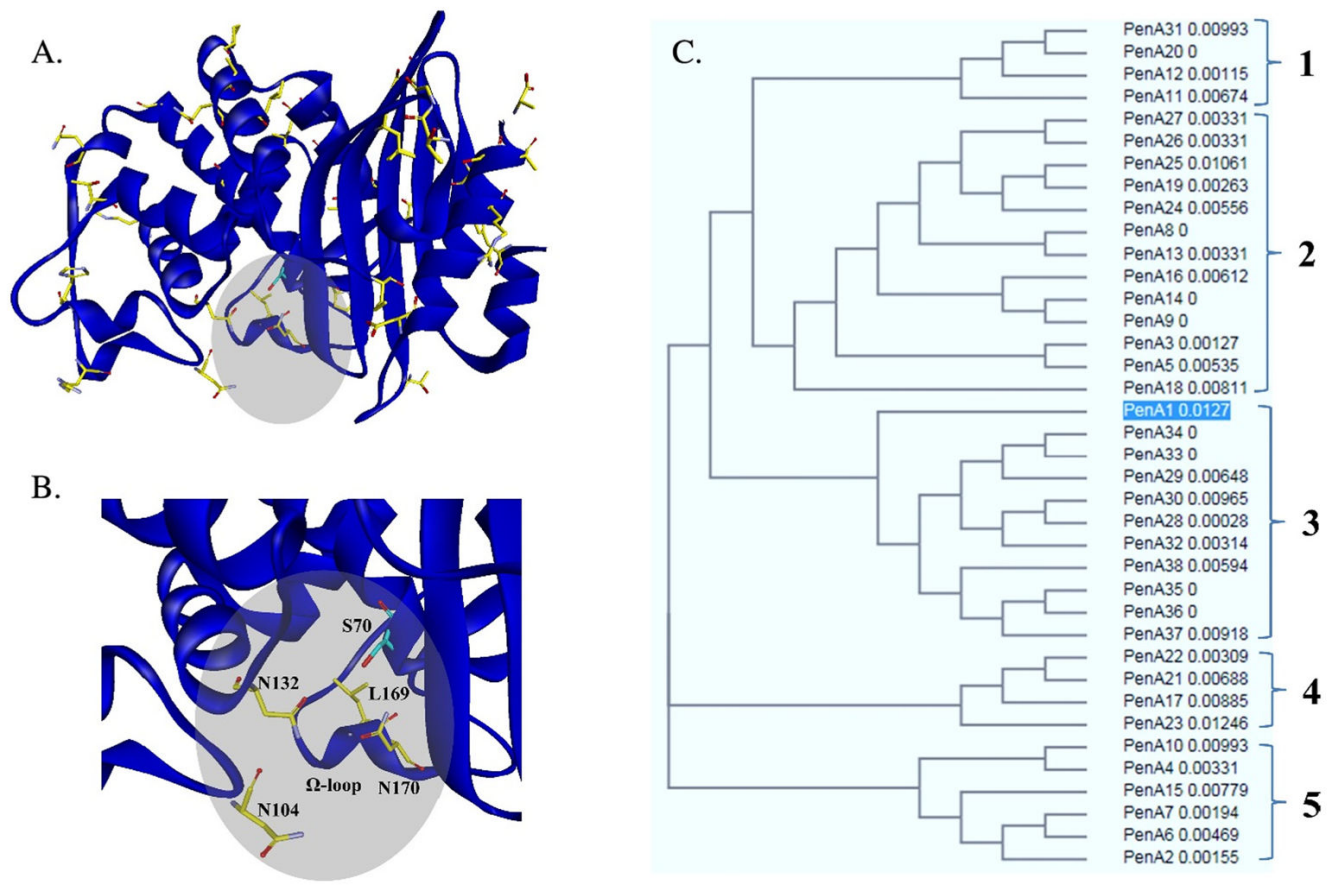
A, PenA crystal structure representing residues (yellow sticks) that are variable in the different clinical isolates. A gray circle highlights the location of the active site, catalytic S70 (cyan sticks). Bg, Enlarge view of the PenA active site revealing the active site residues (N104, N132, L169, and N170) that possess amino acid substitutions in the PenA variants. C, Phylogenetic tree constructed using Clustal Ω reveals the presence of 5 clades; PenA1 is highlighted in blue (Li et al., 2015; McWilliam et al., 2013; Sievers et al., 2011)

To determine the primary amino acid sequence diversity between PenA’s in *B. multivorans*, we compared the PenA amino acid sequences from 48 different clinical isolates; 37 novel PenA variants were identified (Table 2). PenA variants possessed 5 to 17 different amino acid substitutions (amino acid numbering is based on the Ambler system; Ambler, 1980) compared with PenA from *B. multivorans* ATCC 17616, the strain in which PenA was first described (Prince et al., 1988). The locations of these amino acid substitutions were mapped to the PenA crystal structure (Fig. 1A). Several amino acid substitutions (N104S, N132S, L169P, and N170K) were found within the motifs associated with the active site (Fig. 1B). These amino acid substitutions are likely to alter the activity of PenA. Previously, amino acid substitutions at position L169 in PenA were shown to possess enhanced ceftazidime resistance (Papp-Wallace et al., 2017a; Yi et al., 2012). Moreover, substitutions at residues N104, N132, and N170 in other class A β-lactamases were shown to influence either the binding of β-lactams and/or β-lactamase inhibitors as well as the turnover or inhibition (Frase et al., 2011; Ourghanlian et al., 2017; Ramdani-Bouguessa et al., 2011).

The 37 variants clustered into ~5 clades based on phylogenetic analysis (Fig. 1C). The N189S and S286I amino acid substitutions were found in all PenA variants. Other prevalent amino acid substitutions included V247A and T267A present in 92% and 73% of the variants, respectively. The largest cluster (2) consisted of the 13 variants with a high occurrence of T19A, H60Y, and G77A substitutions (Fig. 1C and Table 2). The second largest group (3) possessed the most (i.e., 12–17) amino acid substitutions in PenA. The majority of these variants carried the T3A, L10V, N104S, and R141L amino acid substitutions, as well as T227A and A280T, which were not present in the other 4 subgroups. The remaining 3 clades contained 4–6 variants; however, the differentiating factor between them included the occurrence of T19A, T52A, and/or S25L in group 1 and P201X and/or G228A in group 4. Clade 5 mostly possessed the prevalent amino acid substitutions mentioned above with a few others interspersed.

### 3.2. Identification of a PenA peptide for antibody production

**Fig. 2:**
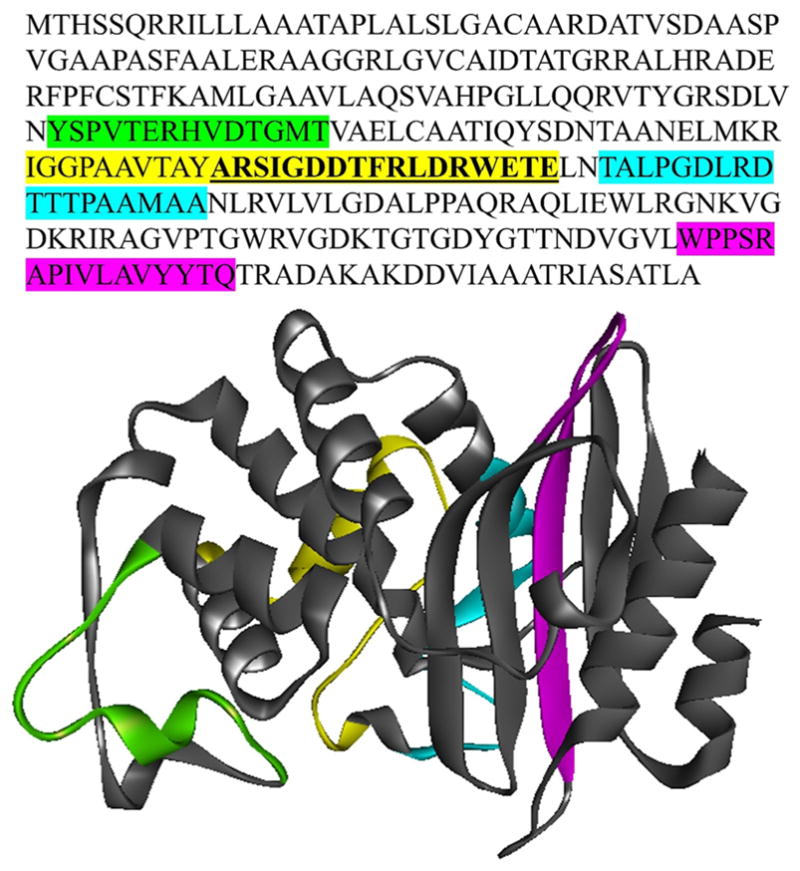
Based on the amino acid sequence variability observed with PenA in the 50 clinical isolates (Table 2), 4 regions (highlighted in green, yellow, cyan, and magenta) were identified within the PenA amino acid sequence that did not possess any substitutions (top); regions are mapped onto the PenA proteins structure (below). NEP conducted further analysis and found that the yellow region (bold and underlined) would make the most favorable antigen.

Due to the significant heterogeneity in amino acid sequence, the polyclonal anti-PenA antibody that we had generated previously (Papp-Wallace et al., 2017a) using the full-length purified PenA β-lactamase as the antigen was unable to recognize the PenA variants in the clinical isolates via immunoblotting. Thus, we set out to generate a better anti-PenA antibody. Based on the 37 different PenA amino acid sequences, we identified conserved regions within the protein (Fig. 2). Four regions of PenA were found to not possess any amino acid substitutions. With additional guidance provided by NEP, a single PenA-peptide (CARSIGDDTFRLDRWETE; the first amino acid of this peptide, tyrosine [Y] was replaced by cysteine [C]) was chosen for polyclonal antibody production in rabbits (Fig. 2).

### 3.3. The polyclonal anti–PenA-peptide antibody possesses a low limit of detection

**Fig. 3:**
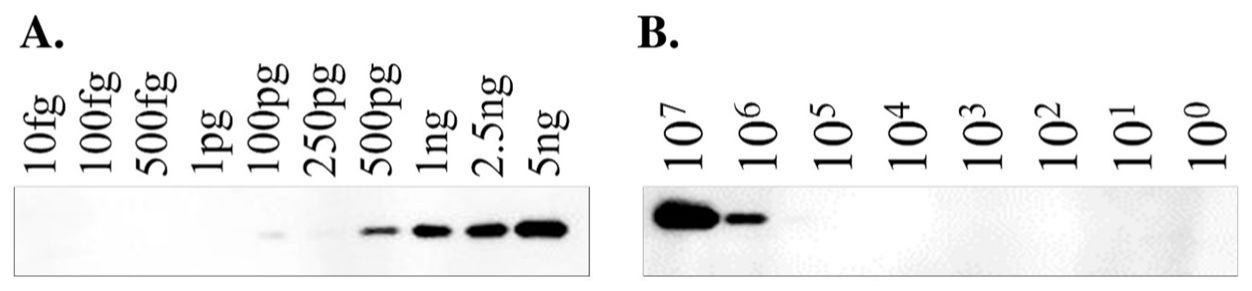
Determining the sensitivity of the anti–PenA-peptide antibody. A, Immunoblot using decreasing concentrations of purified PenA β-lactamase. B, Immunoblot using decreasing CFUs of *B. multivorans* ATTC 17616 after induction with 1 μg/mL of imipenem.

To determine the limits of detection for the anti–PenA-peptide antibody, decreasing concentrations (10 fg–5 ng) of purified PenA protein were used for an immunoblot. The antibody could distinguish small amounts of protein, and a band for 500 pg of purified PenA was easily detected; moreover, a very weak band was observed at 100 pg (Fig. 3A). Our previous polyclonal anti-PenA antibody could only recognize 250 ng of purified PenA protein (data not shown). Thus, the anti–PenA-peptide antibody discriminates 200–500× better than the former anti-PenA antibody. The detection limit of the antibody was further assessed using *B. multivorans* ATCC 17616 after induction of *bla*_PenA_ using imipenem. The anti–PenA-peptide antibody could detect PenA protein using ~10^6^ CFUs (Fig. 3B).

### 3.4. The polyclonal anti–PenA-peptide antibody detects PenA in 50 different B. multivorans clinical isolates

**Fig. 4:**
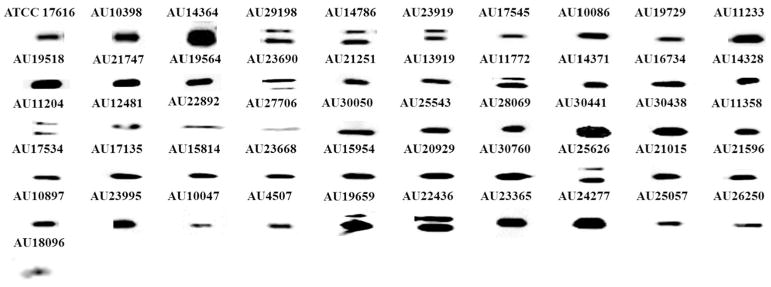
Immunoblot on crude extracts of 50 different clinical isolates of *B. multivorans* after induction with 1 μg/mL of imipenem. Strains AU25626 and AU21251 were not sequenced, but are presented in the immunoblot.

To test the ability of the anti–PenA-peptide antibody to detect different variants of PenA, 50 *B. multivorans* clinical isolates were grown to log phase, induced with imipenem, and prepared for immunoblotting. The anti–PenA-peptide antibody was able to detect all 37 PenA variants within the 50 isolates (Fig. 4). A nonspecific higher-molecular-weight band was observed in some of the isolates (e.g., AU29198, AU14786, and AU22436); studies are underway to identify this protein. We speculate that the higher-molecular-weight band represents unprocessed PenA protein prior to cleavage of the signal peptide; PenA’s signal peptide is 27 amino acids and 2.7 kDa.

### 3.5. The polyclonal anti–PenA-peptide antibody detects other Pen-like β-lactamases within Bcc

**Fig. 5:**
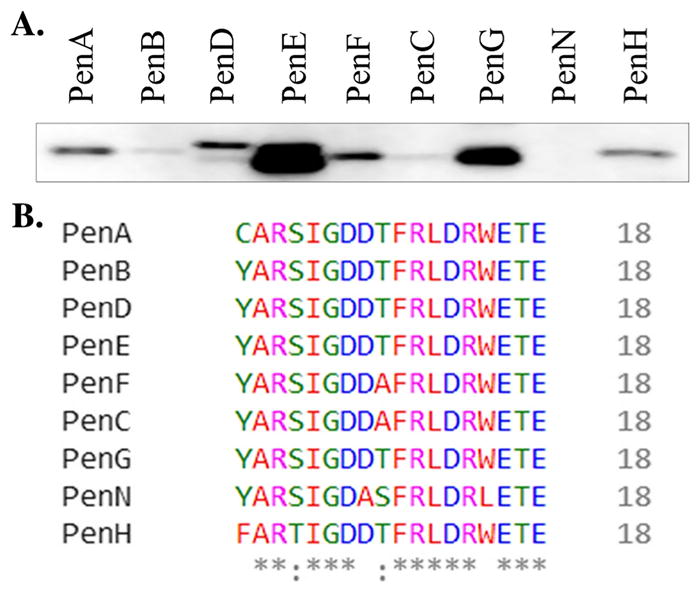
Assessing the cross-reactivity of anti–PenA-peptide antibody against *Burkholderia* spp. A, Immunoblot on *B. multivorans* ATTC 17616 *bla*_PenA_, *B. cenocepacia* AU0583 *bla*_PenB_, *B. pyrrocinia* AU1114 *bla*_PenD_, *B. vietnamiensis* AU3578 *bla*_PenE_, *B. ambifaria* AU5203 *bla*_PenF_, *B. stabilis* AU9035 *bla*_PenC_, *B. dolosa* AU9336 *bla*_PenG_, *B. gladioli* AU1009 *bla*_PenN_ and *B. ubonensis* AU7314 *bla*_PenH_ grown to log phase, induced with 1 μg/mL of imipenem, and prepared as crude extracts. B, Clustal Ω sequence alignment of the peptides from the different Pen-like β-lactamases compared with the PenA peptide used for antibody production.

The cross-reactivity of the PenA-peptide antibody was assessed against other Pen-like β-lactamases (PenB, PenC, PenD, PenE, PenF, PenG, PenH, and PenN) produced by *Burkholderia* species. Interestingly, among the clinical isolates tested using ~10^8^ CFUs, the antibody detected PenB, PenD, PenE, PenF, PenC, PenG, and PenH, which are produced by strains within the Bcc (Fig. 5A). The PenE and PenG β-lactamases of *B. vietnamiensis* and *B. dolosa*, respectively, demonstrated the most intense bands. The PenN β-lactamase of *B. gladioli*, which is not a member of the Bcc, was not within the limit of detection. Comparison of the analogous peptide sequences from the different Pens revealed that the PenN peptide possessed the most sequence diversity with 4-amino-acid substitutions compared with the peptide used for antibody production (Fig. 5B).

## 4. Conclusions

Here, we document the significant sequence heterogeneity in the PenA carbapenemase based on the analysis of 48 different clinical strains of *B. multivorans*. Thirty-seven novel PenA variants were identified with 5–17 different single amino acid substitutions. Most of the amino acid changes are not to active site residues; thus, we hypothesize that they may increase the stability of the β-lactamase. The impact is currently under investigation.

## Supplementary Material

Supplemental

## Figures and Tables

**Table 1:** The Pen family of *Burkholderial* class A β-lactamases.

Bacterial species (complex)	Pen-like β-lactamase	Reference
*Burkholderia multivorans* (Bcc)	PenA	(Trepanier et al., 1997)
*Burkholderia cenocepacia* (Bcc)	PenB	(Poirel et al., 2009)
*Burkholderia stabilis* (Bcc)	PenC	(Poirel et al., 2009)
*Burkholderia pyrrocina* (Bcc)	PenD	(Poirel et al., 2009)
*Burkholderia vietnamiensis* (Bcc)	PenE	(Poirel et al., 2009)
*Burkholderia ambifaria* (Bcc)	PenF	(Poirel et al., 2009)
*Burkholderia dolosa* (Bcc)	PenG	(Poirel et al., 2009)
*Burkholderia ubonensis* (Bcc)	PenH	(Poirel et al., 2009)
*Burkholderia pseudomallei* (Bpc)	PenI	(Poirel et al., 2009)
*Burkholderia oklahomensis* (Bpc)	PenJ	(Poirel et al., 2009)
*Burkholderia mallei* (Bpc)	PenK	(Poirel et al., 2009)
*Burkholderia thailandensis* (Bpc)	PenL	(Poirel et al., 2009)
*Burkholderia humptydooensis* (Bpc)	PenM	This study
*Burkholderia gladioli*	PenN	This study

**Table 2:** Amino acid comparison of the PenA variants sequenced in clinical isolates of *B. multivorans* to *B. multivorans* ATCC 17616; (L), correspond to amino acid substitutions in the PenA leader peptide; and amino acid numbering is based on the Ambler system (Ambler, 1980).

Strain	Amino acid substitutions, insertions, or deletions present	No. of Δs	PenA allele	PenA RefSeq accession	GenBank genome accession
ATCC 17616, AU21747		0	PenA1	WP_012216561.1	NC_010086, PVGM00000000
AU17545, AU19729	S(L5)P, N189S, V247A, T267A, S286I	5	PenA2	WP_105796499.1	PVGB00000000, PVGH00000000
AU11233	T19A, N189S, V247A, T267A, S286I	5	PenA3	WP_105769622.1	PVFL0000000
AU26250, AU15954, AU22892	H60Y, N189S, V247A, T267A, S286I, A290G	6	PenA4	WP_105781374.1	PVGY00000000, PVFW00000000, PVGO00000000
AU10398	S(L5)P, T19A, N170K, N189S, V247A, T267A, S286I	7	PenA5	WP_105822028.1	PVFH00000000
AU19518, AU28069	S(L5)P, N189S, A205T, G228A, V247A, T267A, S286I	7	PenA6	WP_105803562.1	PVGD00000000, PVHB00000000
AU14371, AU10897	S(L5)P, N189S, A205T, V247A, T267A, S286I, A290G	7	PenA7	WP_105758546.1	PVFT00000000, PVFI00000000
AU14786	S(L5)P, T19A, G77A, N189S, V247A, T267A, S286I	7	PenA8	WP_048804470.1	PVFU00000000
AU25543	T19A, G77A, A86E, N189S, P201A, V247A, T267A, S286I	8	PenA9	WP_105772526.1	PVGX00000000
AU19564	A30V, N189S, V192M, V247A, T267A, S286I, A287S, A290G	8	PenA10	WP_105846710.1	PVGE00000000
AU13919, AU14328	T19A, F34L, N189S, A205T, V247A, T267A, S286I, A290G	8	PenA11	WP_105777201.1	PVFQ00000000, PVFR00000000
AU23995	T19A, S25L, T52A, N189S, V247A, T267A, S286I, A290G	8	PenA12	WP_105809721.1	PVGT00000000
AU11772, AU23919	S(L5)P, T19A, P67R, G77A, N189S, V247A, T267A, S286I	8	PenA13	WP_105766562.1	PVFN00000000, PVGS00000000
AU18096	T19A, G77A, A86E, N189S, P201A, V247A, T267A, S286I	8	PenA14	WP_105772526.1	PVGC00000000
AU21015	S(L5)P, P26R, N189S, D239A, V247A, T267A, S286I, A290G	8	PenA15	WP_107999608.1	PZZC00000000
AU30760	T19A, H60Y, G77A, N189S, P201A, V247A, T267A, S286I, A290G	9	PenA16	WP_105825204.1	PVHJ00000000
AU4507	A24P, A30S, N189S, P201H, G228A, V247A, T267A, S286I, A290G	9	PenA17	WP_105951298.1	PVHL00000000
AU24277	S(L5)P, A15T, T19A, N189S, P201L, V247A, T267A, S286I, A290G	9	PenA18	WP_105835300.1	PVGU00000000
AU12481	T19A, A30S, H60Y, G77A, N189S, V247A, T267A, S286I, A290P	9	PenA19	WP_105791036.1	PVFO00000000
AU30441	T19A, S25L, T52A, G77A, N189S, V247A, T267A, S286I, A290G	9	PenA20	WP_088926609.1	PVHH00000000
AU20929	S(L5)P, A23T, N189S, P201A, I208S, G228A, V247A, T267A, S286I, A290G	10	PenA21	WP_105841035.1	PVGJ00000000
AU29198	S(L5)P, ΔA29, A30L, N189S, P201A, G228A, V247A, T267A, S286I, A290G	10	PenA22	WP_105842598.1	PVHD00000000
AU17534	T19A, A30S, Q92R, N189S, Q206R, G228A, V247A, T267A, S286I	10	PenA23	WP_105782476.1	PVGA00000000
AU23668	T(L2)P, S(L5)P, T19A, A30S, H60Y, G77A, N189S, V247A, T267A, S286I	10	PenA24	WP_105813799.1	PVGQ00000000
AU21596	T19A, A30S, T52A, H60Y, G77A, N189S, V247A, T267A, D276E, S286I	10	PenA25	WP_105854385.1	PVGL00000000
AU14364	S(L5)P, T19A, A23T, H60Y, D63G, G77A, L169P, A184E, N189S, V247A, T267A, S286I	12	PenA26	WP_105765688.1	PVFS00000000
AU15814	S(L5)P, T19A, A23T, H60Y, D63G, G77A, N132S, A184E, N189S, V247A, T267A, S286I	12	PenA27	WP_105765076.1	PVFV00000000
AU17135, AU10047	T3A, L10V, T19A, A58T, N104S, R141L, N189S, P201A, V247A, A280T, S286I, A290G	12	PenA28	WP_088924033.1	PVFZ00000000, PVFE00000000
AU16734	T3A, L10V, T19A, R99Q, N104S, R141L, N189S, P201A, T227A, A280T, S286I, A290G	12	PenA29	WP_105792310.1	PVFY00000000
AU19659	T3A, L10V, P26A, A58T, N104S, R141L, N189S, P201A, K219R, A280T, S286I, A290G	12	PenA30	WP_105807557.1	PVGF00000000
AU10086	T19A, S25L, T52A, G77A, H112Y, T118A, N189S, V247A, T267A, R283Q, S286I, A290G	12	PenA31	WP_039217008.1	PVFF00000000
AU23690, AU11358, AU11204	T3A, L10V, T19A, A58T, N104S, R141L, N189S, P201A, T227A, V247A, A280T, S286I, A290G	13	PenA32	WP_105762373.1	PVGR00000000, PVFM00000000, PVFK00000000
AU23365	T3A, L10V, T19A, N104S, R141L, N189S, P201A, T227A, V247A, L250M, A280T, S286I, A290G	13	PenA33	WP_069220914.1	PVGP00000000
AU27706	T3A, L10V, T19A, N104S, R141L, N189S, insertion VL 191–192, P201A, T227A, V247A, L250M, A280T, S286I, A290G	15	PenA34	WP_105856053.1	PVHA00000000
AU30050	T3A, L10V, T19P, V20T, S21D, D22N, V27G, N104S, R141L, N189S, P201A, T227A, V247A, A280T, S286I, A290G	16	PenA35	WP_105795180.1	PVHF00000000
AU30438	T3A, L10V, T19P, V20T, S21D, D22N, V27G, N104S, R141L, N189S, P201A, T227A, V247A, T267A, S286I, A290G	16	PenA36	WP_105795180.1	PVHG00000000
AU22436	T3A, L10V, T19P, V20T, S21D, D22N, V27G, A58T, N104S, R141L, N189S, P201A, T227A, L250M, A280T, S286I, A290G	17	PenA37	WP_088929369.1	PVGN00000000
AU25057	T3A, L10V, G11C, T19P, V20T, S21D, D22N, V27G, N104S, R141L, N189S, P201A, T227A, V247A, A280T, S286I, A290G	17	PenA38	WP_105837729.1	PVGW00000000
