# Supplementary material for: Sequence heterogeneity of the PenA carbapenemase in clinical isolates of Burkholderia multivorans
Source: Diagn Microbiol Infect Dis. Author manuscript; Available in PMC 2019 Nov 1. (PMC6173980; doi:10.1016/j.diagmicrobio.2018.06.005)
Supplement: Supplemental [file NIHMS981107-supplement-Supplemental.pdf]

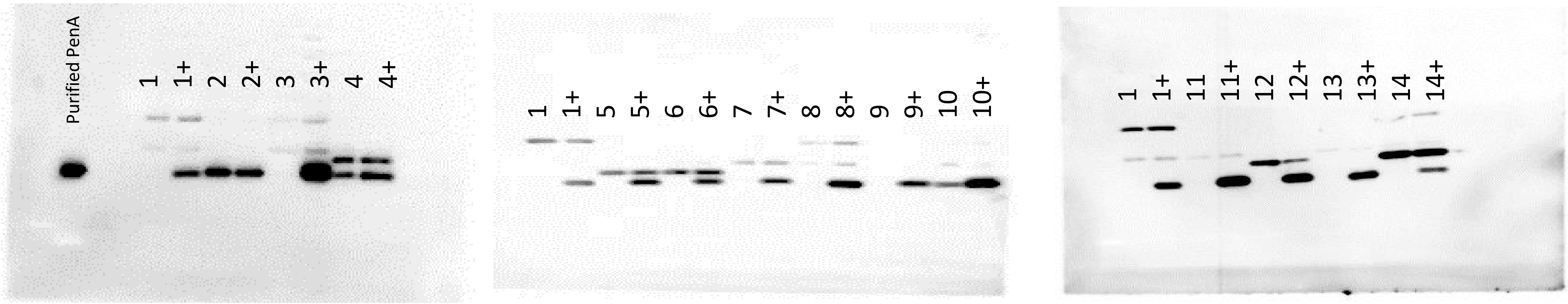

Key: (+) induced with 1 µg/ml imipenem for 2hrs

1 ATCC 17616
2 AU10398
3 AU14364
4 AU29198
5 AU14786
6 AU23919
7 AU17545
8 AU10086
9 AU19729
10 AU11233
11 AU19158
12 AU21747
13 AU19654
14 AU23690

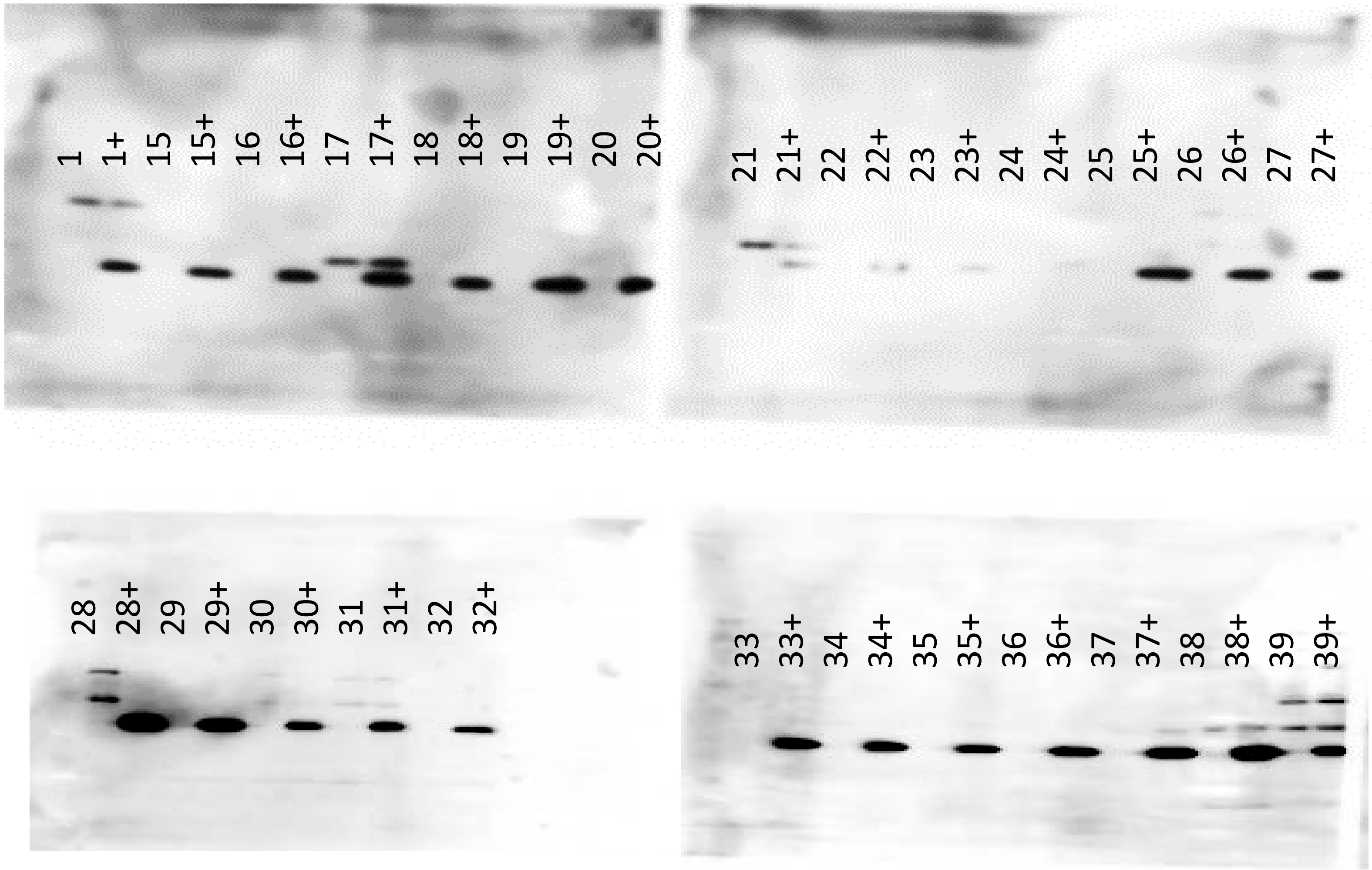

Key: (+) induced with 1 μg/ml imipenem for 2hrs

1 ATCC 17616
15 AU21251
16 AU13919
17 AU11772
18 AU14371
19 AU16734
20 AU14328
21 AU11204
22 AU12481
23 AU22892
24 AU27706
25 AU30050
26 AU25543
27 AU28069
28 AU30441
29 AU30438
30 AU11358
31 AU17534
32 AU17135
33 AU19564
34 AU15814
35 AU23668
36 AU15954
37 AU20929
38 AU30760
39 AU25626

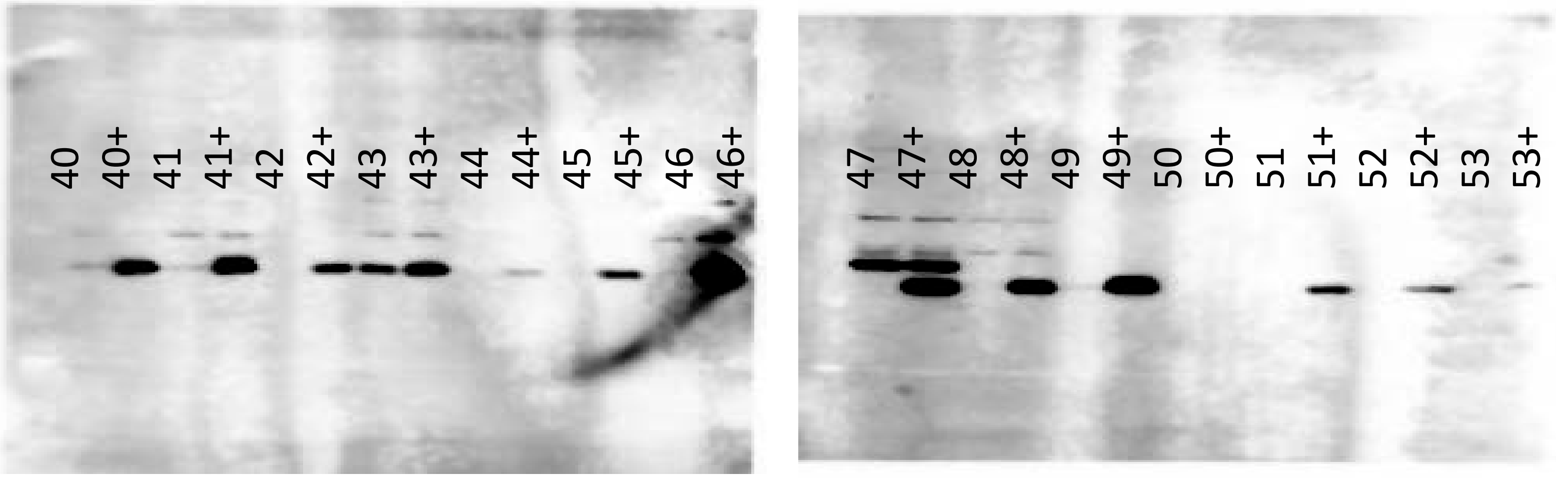

Key: (+) induced with 1 µg/ml imipenem for 2hrs

40 AU21015
41 AU21596
42 AU10897
43 AU23995
44 AU10047
45 AU4507
46 AU19659
47 AU22436
48 AU23365
49 AU24277
50 AU24362 determined to be *B. cenocepacia* (not included in paper)

51 AU25057
52 AU26250
53 AU18096

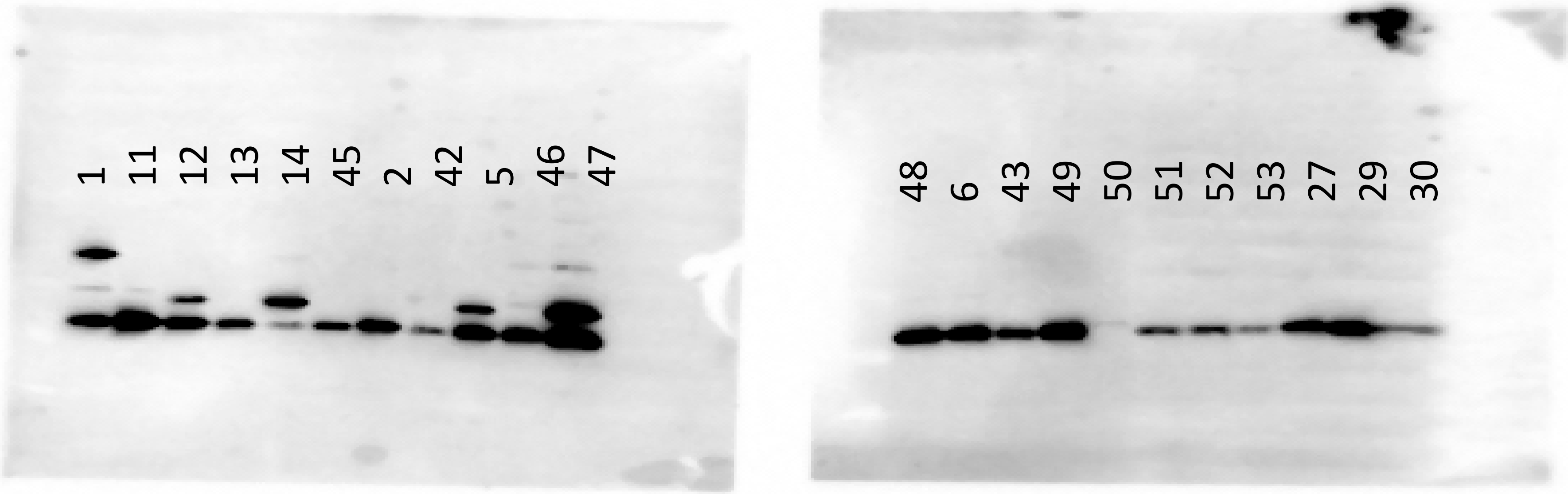

Key: induced with 1 µg/ml imipenem for 2hrs

1. ATCC 17616
11. AU19518
12. AU21747
13. AU19654
14. AU23690
45. AU4507
2. AU10398
42. AU10897

5. AU14786
46. AU19659
47. AU22436
48. AU23365
6. AU23919
43. AU23995
49. AU24277
50. AU24362 (*B. cenocepacia*)

51. AU25057
52. AU26250
53. AU18096
27. AU28069
29. AU30438
30. AU11358

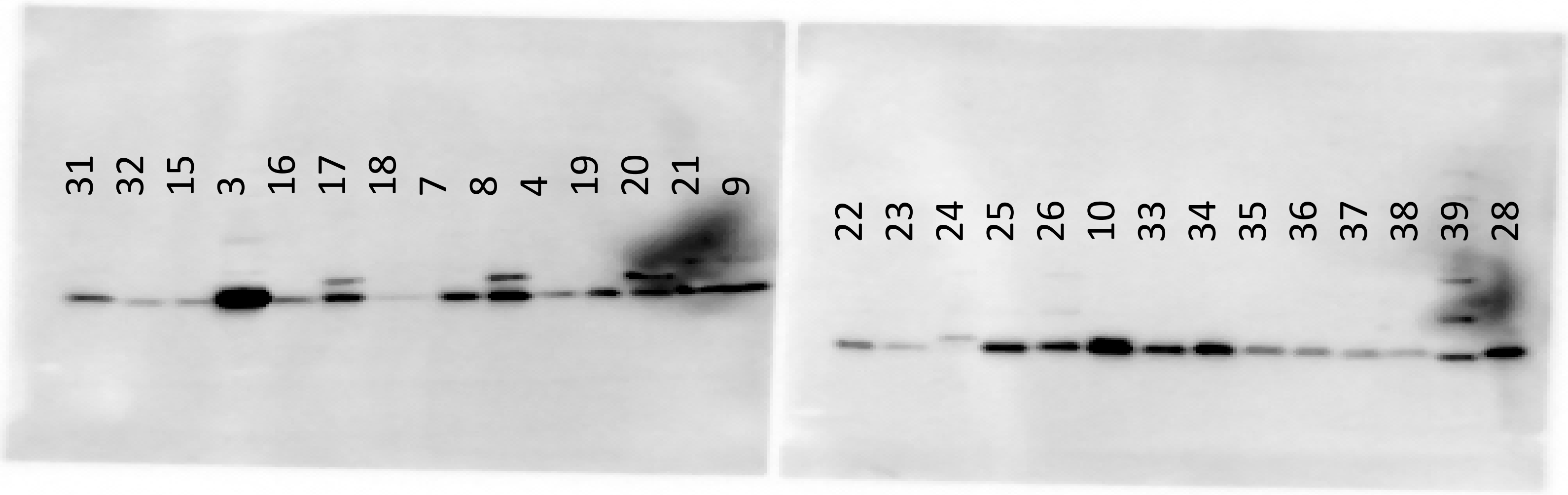

Key: induced with 1 µg/ml imipenem for 2hrs

31. AU17534
32. AU17135
15. AU21251
3. AU14364
16. AU13919
17. AU11772
18. AU14371
7. AU17545
8. AU10086
4. AU29198
19. AU16734
20. AU14328
21. AU11204
9. AU19729
22. AU12481
23. AU22892
24. AU27706
25. AU30050
26. AU25543
10. AU11233
33. AU19564
34. AU15814
35. AU23668
36. AU15954
37. AU20929
38. AU30760
39. AU25626
28. AU30441

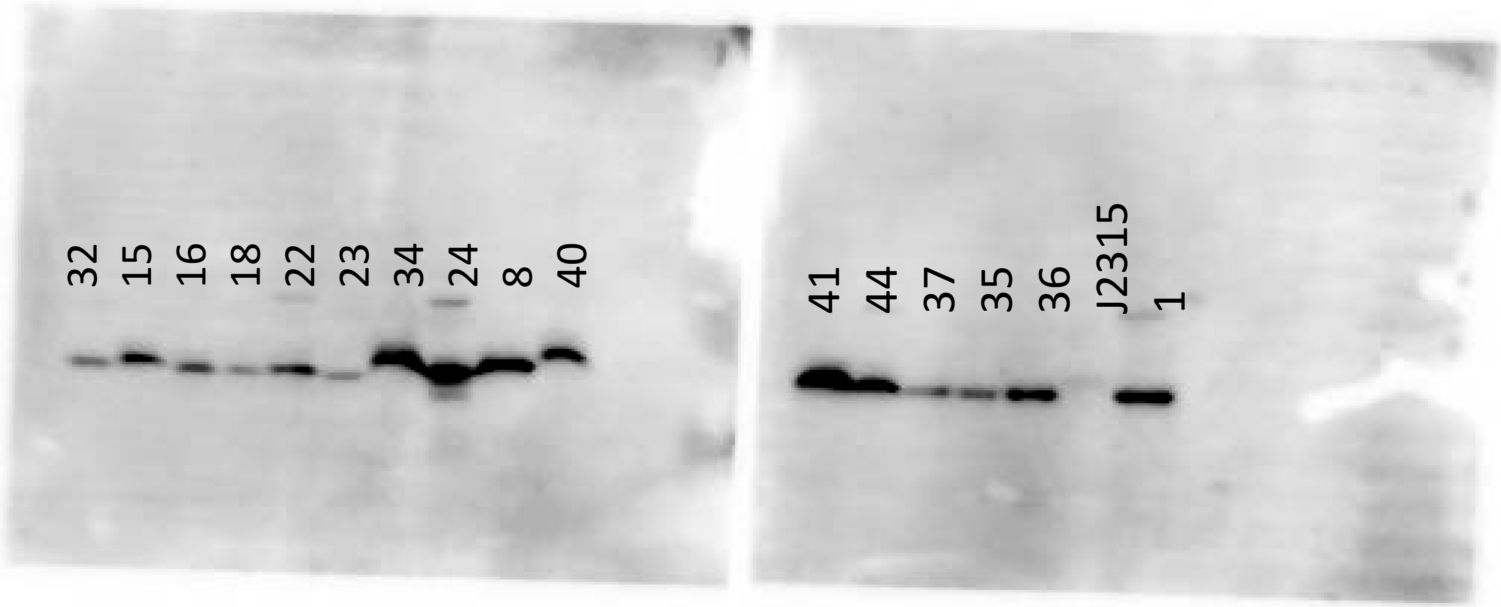

Key: induced with 1 µg/ml imipenem for 2hrs

32. AU17135
15. AU21251
16. AU13919
18. AU14371
22. AU12481
23. AU22892
34. AU15814
24. AU27706

8. AU10086
40. AU21015
41. AU21596
44. AU10047
37. AU20929
35. AU23668
36. AU15954
J2315 (B. cenocepacia)
1. ATCC 17616
